# Trends in the incidence of attention deficit hyperactivity disorder in children and early risk factors

**DOI:** 10.1002/jcv2.70067

**Published:** 2025-11-12

**Authors:** Marika Leppänen, Miika Vuori, Bernd Pape, Anniina Kaittila, Siiri‐Liisi Kraav, Tommi Tolmunen, Merja Anis, Max Karukivi, Päivi Rautava

**Affiliations:** ^1^ Department of Psychiatric and Public Health University of Turku Turku University Hospital Turku Finland; ^2^ Research Unit Social Insurance Institution of Finland Helsinki Finland; ^3^ Department of Mathematics and Statistics University of Vaasa Vaasa Finland; ^4^ Department of Biostatistics University of Turku Turku University Hospital Turku Finland; ^5^ Department of Social Sciences University of Turku Turku Finland; ^6^ Department of Social Sciences University of Eastern Finland Kuopio Finland; ^7^ Department of Adolescent Psychiatry Faculty of Health School of Medicine University of Eastern Finland Kuopio University Hospital Kuopio Finland; ^8^ Department of Social Science University of Turku Turku Finland; ^9^ Department of Adolescent Psychiatry University of Turku Turku University Hospital Turku Finland; ^10^ Research Services Turku University Hospital Public Health University of Turku Turku Finland

**Keywords:** attention deficit hyperactivity disorder, diagnosis, epidemiology

## Abstract

**Background:**

Few studies have used nationwide registry data from both primary and secondary healthcare services to examine the incidence of attention‐deficit/hyperactivity disorder (ADHD) in children while accounting for early risk factors. We aimed to investigate trends in ADHD diagnoses and associated risk factors.

**Methods:**

This study utilized data from population‐based registers, comprising children born between 2001 and 2006 (*N* = 341,632), with follow‐up data available up to age 12. Children who died perinatally, those with unclear or very low (<32 weeks) gestational age, as well as those with severe congenital syndromes or severe or unclear cognitive impairments were excluded. The main outcome was the cumulative incidence of ADHD, defined by ICD‐10 codes F90, F90.0, F90.1, F90.8, F90.9, or F98.8. This outcome was adjusted for gestational age, sex, birth cohort, and maternal psychosocial factors during the child's first 3 years.

**Results:**

The cumulative incidence of ADHD diagnosis at 0–12 years was 4.0% (3.9−4.0%) for the entire study (*N* = 324,766), 6.4% (6.2−6.5%) for boys, and 1.5% (1.5−1.6%) for girls. An ADHD diagnosis was more likely (adjusted odds ratios [95% confidence intervals]) among boys (4.6 [4.3–4.8]) and moderately preterm children (1.3 [1.2–1.4]), as well as in cases where the mother smoked during pregnancy (2.0 [1.9–2.1]), had a psychiatric disorder (2.0 [1.9–2.1]), or lived alone (1.4 [1.3–1.5]), compared to opposite condition. The cumulative incidence of ADHD diagnoses increased over time in both primary and secondary healthcare settings. There was a slight increase in the number of mothers diagnosed with any psychiatric disorder across cohorts, *p* < 0.0001.

**Conclusion:**

The cumulative incidence of ADHD diagnoses in healthcare has been increasing. While psychosocial risk factors are associated with an elevated risk of receiving an ADHD diagnosis, other factors may also contribute to the rising diagnostic rates.

## Key Points

**What's Known**

- ADHD incidence is rising, with both biological and environmental factors contributing to diagnosis. Various levels of the healthcare system, including primary and secondary care, contribute to the diagnostic process.

**What's New**

- Using data from six birth cohorts that included both primary and secondary healthcare records, the incidence of ADHD diagnoses increased notably in boys, approaching the upper limit of established benchmark estimates, while the incidence in girls remained comparatively low. Maternal psychiatric disorders and adverse psychosocial conditions during pregnancy were associated with increased risk of an ADHD diagnosis.

**What's Relevant**

- There is a need to prevent misdiagnoses in boys and improve the recognition of ADHD in girls; careful diagnostic practices and adequate resources across all healthcare sectors may help achieve this.

## INTRODUCTION

Attention deficit hyperactive disorder (ADHD) is an early‐emerging and heterogenous neurodevelopmental disorder (Visser et al., 2014; World Health Organization, 2004). Based on systematic reviews of studies published before 2020, the pooled prevalence estimates of ADHD in children and young people vary between 2.2% and 7.8% (Cortese et al., 2023; Polanczyk et al., 2007; Salari et al., 2023; Sayal et al., 2018; Thomas et al., 2015). The marked variety among reported estimates seems to relate to, for example, the timepoint and location at which a study was conducted; the sex and age of the participants; variation in the use of diagnostic manuals/thresholds and statistical analysis methods; and, in community prevalence studies, variety among informants (Cortese et al., 2023; Polanczyk et al., 2007; Salari et al., 2023; Sayal et al., 2018; Thomas et al., 2015).

Symptom‐based surveys regarding ADHD may not differentiate difficult‐to‐diagnose cases with symptoms close to the diagnostic threshold from clinically relevant cases, as clinicians typically do in practice (Faraone, 2024; Faraone et al., 2021; Vogel et al., 2018). Symptom questionnaires alone do not differentiate between the mere presence of symptoms and the presence of harmful symptoms that interfere with social functioning. However, many studies on the pooled prevalence of ADHD in children have included individual studies that rely only on questionnaires or partial diagnosis (Polanczyk et al., 2007; Popit et al., 2024).

Thus, studies that describe the number of ADHD diagnoses made by clinicians in the entire population may provide more exact information, at least about the healthcare system of the country in which a given study was conducted. The number of recorded ADHD cases seems to be increasing, including in Finland (Atladottir et al., 2015; Joelsson et al., 2016; Kazda et al., 2021; Sayal et al., 2018; Westman et al., 2025). The reasons for this are unknown but hypothesized to relate to the increased awareness of ADHD, mismatch between individuals and a changing environment, or changes in diagnostic practices (Eirich et al., 2022; Gyllenberg et al., 2018; Joelsson et al., 2016; Kazda et al., 2021; Polanczyk et al., 2007; Rogers & MacLean, 2023; Rydell et al., 2018; Sayal et al., 2018; Thomas et al., 2015).

Only a few epidemiological studies have focused on recorded ADHD diagnoses in the entire population (Dalsgaard et al., 2020; Westman et al., 2025), and even less attention has been paid to diagnoses made in primary healthcare and potential trends in this regard. In different clinical practice guidelines, the role of primary healthcare in ADHD identification has been described in various ways, i.e., as screener or as part of diagnostic evaluation. The latter challenges the competence of and resources available in primary healthcare (i.e., child specialists may not be available in all areas) (Mayne et al., 2016). This can lead to diagnosis and treatment failures. Therefore, potential changes in the incidence of ADHD in childhood should also be studied separately for primary and secondary healthcare.

Children with ADHD are at risk of underachievement and co‐occurring psychiatric disorders (De Ronda et al., 2023; Spencer et al., 2007). Thus, it is important to explicate the factors associated with ADHD diagnosis to improve prevention, in‐time recognition, and services for children and families, as well as in studying whether these known risk factors for ADHD are increasing. Although the etiology of ADHD is highly genetic (Franke et al., 2012; State, 2010; Thapar et al., 2013; Willoughby et al., 2020), many other factors may be associated with the risk of being diagnosed with ADHD (Faraone, 2024; Huhdanpää et al., 2021; Loewen et al., 2020; Morales et al., 2015; Sciberras et al., 2011; Thapar et al., 2013; Vuori, Martikainen, et al., 2020; Østergaard et al., 2016) The most significant risk factors for ADHD seem to be preterm birth (Halmøy et al., 2012; Sucksdorff et al., 2015; Willoughby et al., 2020) and the male sex (Dalsgaard et al., 2020; Huhdanpää et al., 2021; Joelsson et al., 2016; Mowlem et al., 2019; Sciberras et al., 2011; Sciutto et al., 2004; Spencer et al., 2007; Willoughby et al., 2020). Furthermore, perinatal maternal adversities, especially if they accumulate, may increase the risk of ADHD diagnoses in offspring (Halmøy et al., 2012; Huhdanpää et al., 2021; Johnson et al., 2019; Sciberras et al., 2011; Van Batenburg‐Eddes et al., 2013; Willoughby et al., 2020; Wolford et al., 2017; Østergaard et al., 2016). These associations may differ depending on a child's sex (Bradley et al., 2001; Huhdanpää et al., 2021; Johnson et al., 2019; Knudsen et al., 2021; Mokrova et al., 2010; Morales et al., 2015) and age (Willoughby et al., 2020). Good psychosocial conditions among families may, however, improve ADHD recognition via better access to diagnostics evaluation (Centers for Disease Control and Prevention, 2010; Sayal et al., 2018; Willoughby et al., 2020; Wright et al., 2015).

Our aim was to (1) describe the cumulative incidence of recorded ADHD diagnoses in primary and secondary healthcare among Finnish boys and girls from birth up to 12 years of age; (2) examine and describe factors potentially associated with an ADHD diagnosis, including maternal prenatal psychosocial conditions (psychiatric morbidity, smoking, age, relationship and employment status, and parity) as well as the child's moderate prematurity, sex, and age at first diagnosis; and (3) analyze birth cohort trends to assess potential changes in the cumulative incidence of ADHD diagnoses in primary and secondary healthcare. We hypothesized that the incidence of ADHD diagnoses in healthcare services is increasing, and that the child's age, sex, moderate prematurity, and maternal psychosocial factors are associated with the risk of receiving an ADHD diagnosis.

## MATERIALS AND METHODS

### Study design

This quantitative and retrospective register study utilized the following population‐based registers: The Finnish Medical Birth Register covers all births in Finland, the Register on Congenital Malformations covers all registered congenital malformations, and the Finnish Discharge Register covers over 95% of all public inpatient and outpatient healthcare visits in Finland (Sund, 2012). The Strengthening the Reporting of Observational Studies in Epidemiology cohort study checklist (Vandenbroucke et al., 2014) was used as guidance in reporting this study.

### Study population

All Finnish children (*N* = 341,632) born between January 1, 2001, and December 31, 2006, were included and followed until the end of the year in which they reached an age of 12 years. We excluded infants who died perinatally (stillborn infants and those who died ≤7 days after birth), children with unclear or very low (<32 weeks) gestational age, congenital malformations, and syndromes classified as severe by the register holder as well as those with severe, profound, other or unclear cognitive impairments defined with diagnosis codes F72−F73 and F78−F79 according to the International Classification of Diseases, 10^th^ Revision (ICD‐10) because of the potential confounding effect on the studied outcome. All mothers (*N* = 240,020) of the study children were included in this study.

### ADHD diagnosis as outcome

The Finnish Care Register for Healthcare provided data on ADHD diagnoses for all children within the study population. Diagnoses via primary healthcare have been available since 2011, whereas prior register data included secondary healthcare diagnoses, including inpatient and outpatient visits, since 1969. If a diagnosis was registered via primary healthcare, it was labeled as a primary healthcare diagnosis, and the same was true for secondary healthcare. A new diagnosis was the first diagnosis for a given patient in the register. In this study, we use the term “ADHD” to describe all forms of hyperkinetic and attention deficit disorders in the ICD‐10: F90.0–disturbance of activity and attention, F90.1–hyperkinetic conduct disorder, F90.8–other hyperkinetic disorders, F90.9–hyperkinetic disorder, unspecified, F98.8–other specified behavioral and emotional disorders with onset usually occurring in childhood and adolescence, and F90–a less precise code for ADHD. In Finland, the diagnosis of hyperkinetic disorder is based on the ICD‐10, and the Finnish Current Care Guideline for ADHD (last updates 2019 and 2025; Käypä Hoito, 2019) is followed in diagnostics and assessment. In the Finnish version of the ICD‐10 (THL, 2011), the diagnosis code F98.8 is described as “attention deficit without hyperactivity.” Consequently, from 2001 to 2018, this code may have been used for cases that do not fully meet the ICD‐10 diagnostic criteria for ADHD. Our preliminary analysis indicates that the non‐specific code F90 was occasionally used in primary healthcare, and some children received an ADHD diagnosis before the age of 4. We included these cases to reflect the “real‐world” application of diagnosis codes.

In Finland, primary healthcare is provided in health centers and specialized healthcare facilities, mainly in hospitals (https://www.eu‐healthcare.fi/healthcare‐in‐finland/healthcare‐system‐in‐finland/). Here, we use the term “secondary healthcare” to describe specialized health care, including secondary and tertiary healthcare and both inpatient and outpatient visits. The validity of ADHD diagnoses via secondary healthcare in Finnish children was evaluated in 2011%, and 88% of re‐evaluated cases met the diagnostic criteria based on the Diagnostic and Statistical Manual of Mental Disorders (5th) (Joelsson et al., 2016).

Cumulative incidence was studied for all children and separately for girls and boys and analyzed to determine trends by birth cohort between 2001 and 2006. We also separately studied ADHD diagnoses made at ages 0−7 and >8−12 to assess whether there are differences in associations with risk factors in this regard. We found the highest incidence of ADHD diagnosis at age 8, so we explored the potential differences in children who have received ADHD diagnosis below or above age 8. The incidence of ADHD was studied in association with maternal psychosocial and child‐related factors that have been found to be related to the studied outcome (Johnson et al., 2019; Sucksdorff et al., 2015; Willoughby et al., 2020; Østergaard et al., 2016). As prematurity is one of the strongest risk factors for ADHD, we excluded very preterm infants but included moderately preterm (>32 weeks but <37 weeks of gestation) and full‐term (>37 weeks) infants to concentrate on psychosocial risk factors. Maternal perinatal psychosocial variables (smoking, relationship status, employment status, and whether a mother was a first‐time mother were dichotomous, yes/no), and are described in an earlier study with the same sample (Leppänen et al., 2023). We considered mothers as having some psychiatric disorder if they had received any diagnosis listed in the chapter on mental or behavioral disorders in the ICD‐10 between one year before childbirth and four years after the birth year, thus covering the perinatal period and the early childhood years.

### Statistical analyses

Differences in background variables for children having versus not having received an ADHD diagnosis were assessed with the unpaired *t*‐test if they were continuous and with the chi‐square independence test if they were categorical. We applied Cochran Armitage tests to consider possible time trends in the explanatory variables for ADHD diagnosis over birth cohorts.

Since exact dates of birth were not available, we used logistic regressions rather than time‐to‐event analyses to study the likelihood of receiving an ADHD diagnosis until the age of 12. This was done both overall and separately for the 0−7 and 8−12 age groups. The reference groups in the analyses stratified by age contained both children without ADHD diagnosis and children who had received their ADHD diagnoses while outside the age group in question. The regressors were the year of the birth cohort; gestational age (moderate preterm status); the sex of the child; and the mother's prenatal smoking status, employment status, cohabitation, age, status as a first‐time mother, and psychiatric diagnoses. We also investigated the interactions between sex and the remaining regressors but excluded them from the final analysis, as none ultimately proved to be significant. Only cases with complete data on all covariates entered the regression.

We obtained cumulative incidences by cohort and age by dividing the number of children in the cohort who had already received a diagnosis at the respective age by the number of children originally in the respective cohort. No standardization or weighting schemes were applied, as we collected data from all children born in Finland from 2001 to 2006 and the incidences refer to the population of these cohorts rather than the population of children living in Finland.

To test differences in cumulative incidence trends between birth cohorts, we first created dummy variables for each year of age, indicating whether ADHD has already been diagnosed at that age or not. We then applied generalized estimating equations (GEE) for repeated measures, with a binomial distribution, a logit link function, and an autoregressive working correlation matrix of order one upon those indicators with the birth cohort, age, and the birth cohort × age interaction as explanatory variables. Starting from 3 years of age, we used the SLICE statement in SAS in order to check for differences in cumulative incidence between birth cohorts for each year of age separately (analysis of simple effects). As primary healthcare diagnosis was available was available only from 2011 onwards, we repeated those analyses using secondary healthcare data only. We applied Cochran‐Armitage tests for trend in age‐wise incidence rates over birth cohorts stratified by sex and health care register. We restricted the sample from the primary care register to 10–12 years old children only, since only those had primary care data available for all birth cohorts.

The analyses were carried out using the statistical software SAS for Windows version 9.4 (SAS Institute Inc., Cary, NC, USA). Two‐sided *p*‐values below 0.05 were considered statistically significant.

## RESULTS

### Participants

**FIGURE 1:**
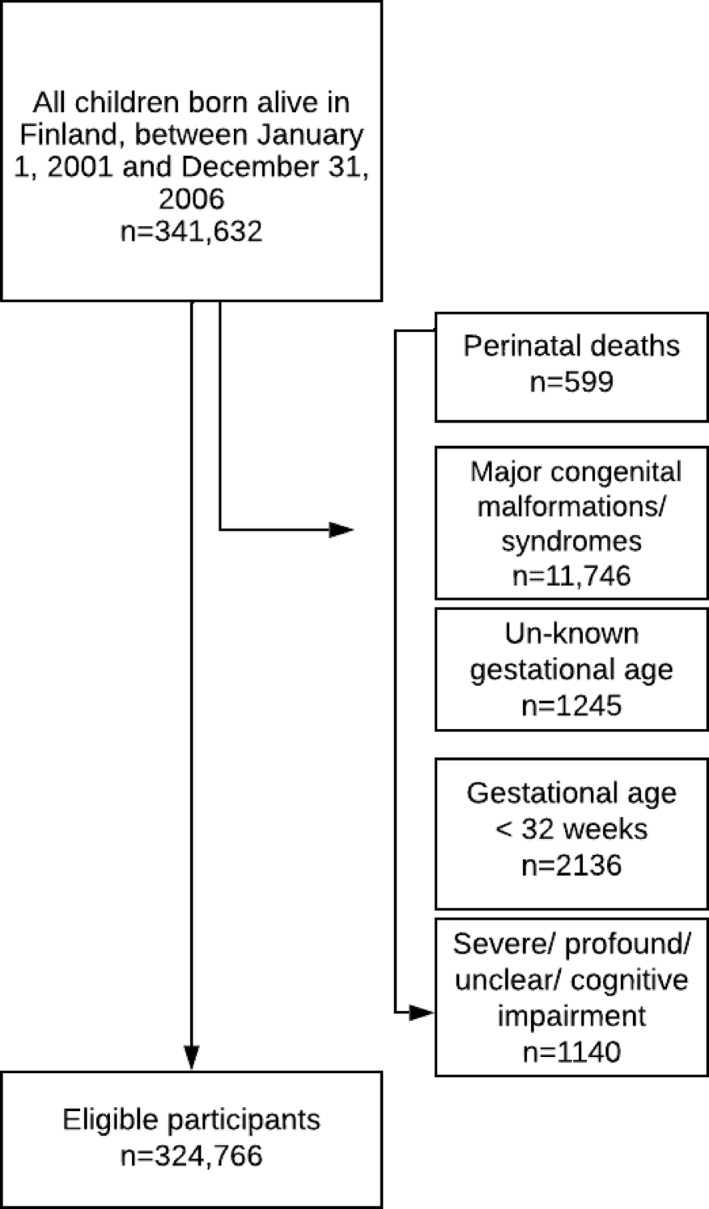
Number of children born in 2001−2006, excluded and included study infants.

Of all the children born from 2001 to 2006, 324,766 children were eligible for the study (Figure 1).

### Descriptive data

Children who had an ADHD diagnosis were more often boys and were born with lower gestational age, and their mothers were more often unemployed, had smoked prenatally, had a psychiatric disorder, lived as single parents, and were younger or first‐time mothers as compared to children without an ADHD diagnosis (Table 1).

**TABLE 1:** Background characteristics of study children.

	Children with ADHD diagnosis (*N* = 12,922)	Children without ADHD diagnosis (*N* = 311,844)	Number of missing covariates
	Mean (minimum−maximum) or percentage (number of observations)		
Mothers			
Age (years)	27.9 (14−48)	29.5 (13−52)	0
First‐time mother	46.8% (6040)	41.7% (130,027)	201
Prenatal smoking	29.2% (3695)	14.5% (44,164)	7876
Single‐parent household	13.0% (1576)	7.4% (22,087)	15,070
Employed	74.5% (8227)	81.0% (219,893)	42,093
Any psychiatric diagnosis	14.3% (1846)	6.3% (19,508)	0
Children			
Gestational age (weeks)	39.7 (32−44)	39.8 (32−45)	0
Moderate preterm	5.9% (763)	4.6% (14,304)	0
Girls	18.7% (2410)	50.3% (156,908)	0
Boys	81.3% (10,512)	49.7% (154,936)	0

*Note*: Covariates that are measured or observed in health care are covered in 100%, but some covariates that have been asked from mothers during pregnancy are not covered fully (smoking habits and information about working status), number of missing is presented above.

In total, 17,594 (7.3%) of studied mothers had a psychiatric disorder diagnosis between one year before the childbirth year and their children reaching the age of 4. The most common diagnostic codes were (F30–F39) mood (affective) disorders (*N* = 9,953, 4.1% of all mothers). The second most common diagnostic codes were (F40–49) anxiety, dissociative, stress‐related, somatoform, and other nonpsychotic mental disorders (*N* = 8,882, 3.7%). There were only 1024 mothers (0.4% of study mothers) who had diagnostic codes (F90−98) behavioral and emotional disorders with onset typically occurring in childhood and adolescence, including ADHD.

Furthermore, all background covariates were analyzed by birth cohort for all study children born from 2001 to 2006. There was no increase or decrease in the percentages of children both preterm versus full‐term or as girls or boys from 2001 to 2006 (*p* > 0.05). The percentage of first‐time mothers increased by one percentage point (41.0% in 2001 and 42.2% in 2006), and mothers with any psychiatric diagnosis (5.5% in 2001 and 7.5% in 2006) increased by two percentage points from 2001 to 2006 (*p* < 0.0001). There were 0.4% points fewer smoking mothers in the 2006 birth cohort than in the 2001 birth cohort (*p* < 0.05). The percentage of mothers who lived with a partner varied over time (*p* < 0.0001) but was the same in the 2001 and 2006 birth cohorts.

### The cumulative incidence of ADHD diagnosis

The cumulative incidence of ADHD diagnosis in all children at 0−12 years was 4.0% (3.9−4.0%; *N* = 12,922), and it was 1.5% (1.5−1.6%; *N* = 2410) in girls and 6.4% (6.2−6.5; *N* = 10,512) in boys. There were 73 children that received ADHD diagnosis below age 4 (0.57% of all children with ADHD diagnosis), see Sensitive Analysis and Supporting Information S1 for more information.

**FIGURE 2:**
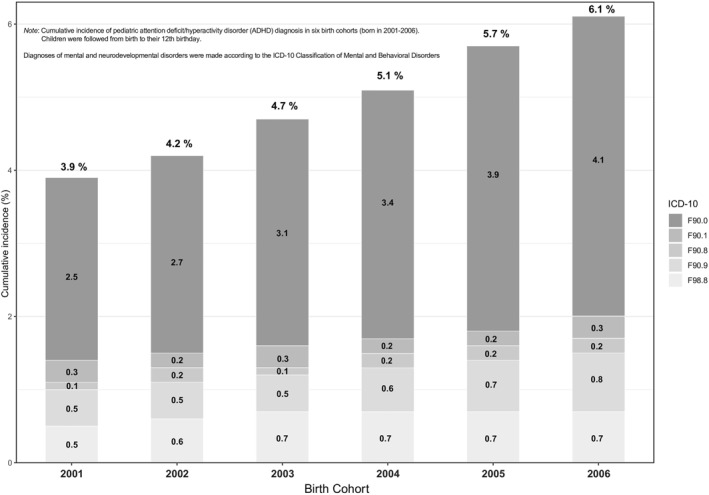
This figure represents the cumulative incidence of attention‐deficit/hyperactivity disorder per birth cohorts between 2001 and 2006 with detailing information of diagnosis codes extracted from health care registers, codes were F90.0 = disturbance of activity and attention (F90 was added to this group), F90.1 = hyperkinetic conduct disorder, F90.8 = other hyperkinetic disorders, F90.9 = hyperkinetic disorder, unspecified, and F98.8 = other, attention deficit without hyperactivity.

We used various ICD‐10 based diagnostic codes to define ADHD, the cumulative incidences were: 3.2% of F90.0 (*N* = 10,379), 0.23% of F90.1 (*N* = 793), 0.16% F90.8 (*N* = 520), 0.6% F90.9 (*N* = 1960), 0.65% F98.8 (*N* = 2108) and 0.07% of unspecified F90 (*N* = 237). Please see details on the proportion of codes for the cumulative incidence of ADHD for each birth cohort in Figure 2.

### The cumulative incidence of ADHD in girls and boys by birth cohort year

**FIGURE 3:**
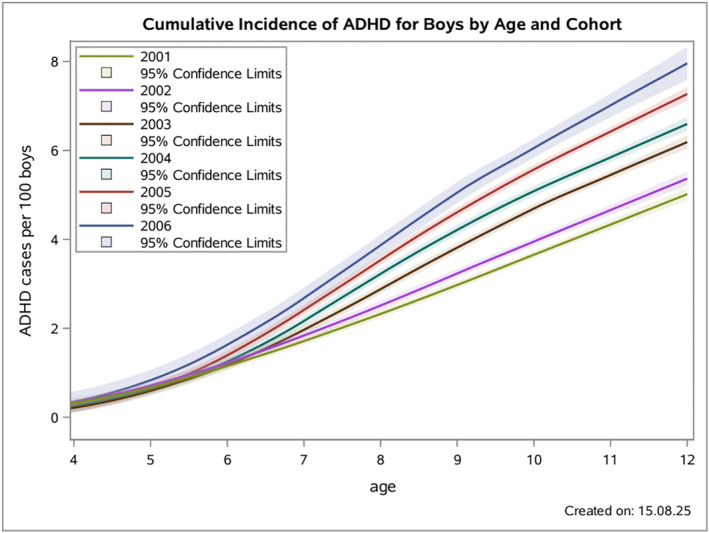
Each birth cohort (2001–2006) was analyzed for the cumulative incidence of attention‐deficit/hyperactivity disorder diagnoses with 95% confidence intervals; the analysis presented here focuses exclusively on boys aged 4–12.

**FIGURE 4:**
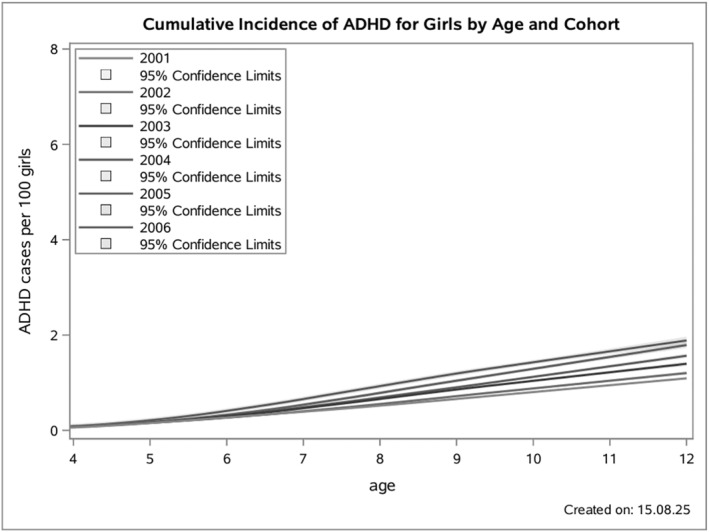
Each birth cohort (2001–2006) was analyzed for the cumulative incidence of attention‐deficit/hyperactivity disorder diagnoses with 95% confidence intervals; the analysis presented here focuses exclusively on girls aged 4–12.

Considering all available ADHD diagnoses from primary and secondary healthcare, the cumulative incidence increased from 5.0% in the 2001 cohort to 7.7% in the 2006 for those born boys and from 1.1% to 1.9% in those born girls. Please see Figures 3 and 4 for the cumulative incidence both boys and girls in all birth cohorts. Birth cohort was associated with an increased risk of receiving an ADHD diagnosis in boys from age 6 to age 12 (*p* < 0.03) and in girls from age 7 to age 12 (*p* < 0.001).

Please see also Supporting Information S1: Table S1 and S2, to receive detailed information on the trend analysis of cumulative incidence for both sexes. There are presented the cumulative incidence (%) of ADHD diagnosis in boys and girls by birth cohort and age and analysis of simple cohort effects for each age from 3 years onwards. Cohort, age, and the cohort × age interaction were all significant for boys (*p* < 0.001). And in girls there was no main effect for birth cohort (*p* = 0.198), but age and the cohort × age interaction were significant (*p* < 0.001).

### Diagnosis in primary and secondary healthcare

While data from the secondary healthcare register was available for all birth cohorts over the whole observation period, the primary healthcare register was not. Consistent with its late start in 2011, we found a shift in distribution of ADHD diagnoses from the secondary to the primary health care sector with increasing birth cohort. The fraction of diagnoses set in primary healthcare alone rose from 14.9% in the 2001 cohort to 28.1% in the 2006 cohort, with a corresponding decrease of diagnoses set in secondary healthcare alone from 68.5% in the 2001 cohort to 42.9% in the 2006 cohort, see Table 2 for further details.

**TABLE 2:** ADHD diagnoses extracted from primary and secondary health care registers.

Birth cohort	Primary health care	Primary and secondary health care^a^	Secondary health care
Number, percentage of diagnoses in the birth cohort, percentage of diagnoses in health care sector groups.			
2001	246, 14.9%, (8.6%)	274, 16.6%, (8.3%)	1131, 68.5%, (16.7%)
2002	291, 16.6%, (10.2%)	366, 20.9%, (11.1%)	1093, 62.5%, (16.2%)
2003	407, 19.8%, (14.3%)	500, 24.3%, (15.1%)	1147, 55.8%, (17.0%)
2004	525, 22.9%, (18.4%)	643, 28.1%, (19.5%)	1123, 49.0%, (16.6%)
2005	633, 25.3%, (22.2%)	746, 29.8%, (22.6%)	1123, 44.9%, (16.6%)
2006	750, 28.1%, (26.3%)	777, 29.1%, (23.5%)	1147, 42.9%, (17.0%)

^a^ ADHD diagnosis was registered both in primary and secondary health care register.

The differential start date of the primary care register also opens up the possibility that the increase of ADHD diagnoses by birth cohort could be a statistical artifact due to late availability of data from that register. We performed the following three robust checks in order to exclude that possibility.

First, we repeated the analysis considering only diagnoses from the secondary care register, which was available for all children over the whole observation period. We found an increase in ADHD diagnoses from 4.3% to 5.5% for boys and from 1.0% to 1.3% for girls across birth cohorts from 2001 to 2006, please see Supporting Information S1: Figure S1 and S2.

Second, we investigated pseudo cumulative incidences using only ADHD diagnoses retrieved from primary healthcare register from age 10 onwards, which is the earliest age for which primary healthcare data was available for all birth cohorts. There was an increase in pseudo cumulative incidence using those diagnoses alone across birth cohorts both overall and for boys and girls separately, with an intermediate peak at the 2005 cohort for girls. Please see detailed information in Supporting Information S1: Table S3.

Finally, we investigated age‐wise incidence rates stratified by sex and health care register, once again disregarding ages below 10 in the primary care register to avoid artifacts from late availability. In all subsets, we found a general trend for increasing incidence rates by birth cohort. The Cochran‐Armitage test for trend rejects the null of no trend in favor of an increasing trend by birth cohort for 10–11 years old boys and for 10–12 years old girls in the primary care register, and for 5‐ and 7 to 12‐ year‐old boys and for 7‐ to 11‐year‐old girls in the secondary care register, please see Supporting Information S1: Table S4–S7.

### Risk factors for an ADHD diagnosis

ADHD was associated with all studied variables that were considered risk factors: sex, low gestational age, and the mother's adverse psychosocial factors (see Table 3). The associations between variables and ADHD diagnosis were slightly stronger when an ADHD diagnosis was made from 0 to 7 than when it was made from 8 to 12. The exception was sex, for which the associations were the same in both age groups.

**TABLE 3:** Background factors and ADHD diagnosis.

	First ADHD diagnosis until the age of 12 years		First ADHD diagnosis at age of 1−7 years		First ADHD diagnosis at age of 8–12 years	
Odds ratio [95% CI]	Unadjusted	Adjusted	Unadjusted	Adjusted	Unadjusted	Adjusted
Mothers (yes vs. no)						
Smoking	2.4 [2.3–2.5]	2.0 [1.9–2.1]	2.5 [2.3–2.6]	2.0 [1.8–2.2]	2.2 [2.1–2.4]	1.9 [1.8–2.0]
Single parenthood	1.8 [1.7–1.9]	1.4 [1.3–1.5]	2.0 [1.8–2.2]	1.5 [1.3–1.6]	1.7 [1.6–1.8]	1.3 [1.2–1.4]
Non‐employed	1.4 [1.4–1.5]	1.1 [1.1–1.2]	1.5 [1.4–1.6]	1.2 [1.1–1.3]	1.4 [1.3–1.5]	1.1 [1.0–1.2]
Psychiatric disorder	2.4 [2.3–2.6]	2.0 [1.9–2.1]	2.7 [2.5–3.0]	2.2 [2.0–2.4]	2.2 [2.1–2.4]	1.8 [1.7–1.9]
First‐time mother	1.2 [1.2−1.3]	1.0 [1.0−1.1]	1.3 [1.2−1.4]	1.1 [1.0−1.2]	1.2 [1.1−1.2]	1.0 [0.9−1.0]
Extra year of age at birth	1.0 [1.0−1.0]	1.0 [1.0−1.0]	1.0 [0.9−1.0]	1.0 [1.0−1.0]	1.0 [1.0−1.0]	1.0 [1.0−1.0]
Children						
Moderate preterm versus term born	1.3 [1.2–1.4]	1.1 [1.2−1.4]	1.5 [1.4–1.8]	1.5 [1.3−1.7]	1.2 [1.1−1.3]	1.1 [1.0–1.3]
Boy versus girl	4.5 [4.3−4.7]	4.6 [4.3–4.8]	4.4 [4.0–4.8]	4.4 [4.0−4.8]	4.4 [4.1−4.7]	4.4 [4.2–4.7]
Cohort	1.1 [1.1−1.1]	1.1 [1.1−1.1]	1.1 [1.1−1.1]	1.1 [1.1−1.1]	1.1 [1.1−1.1]	1.1 [1.1−1.1]

### Sensitive Analyses

As the F98.8 diagnosis code can be used for diagnoses other than attention deficit disorder without hyperactivity, we performed a sensitivity analysis covering cumulative incidence, cohort trends, regressors, univariate and multivariate models. In sensitivity analyses, when F98.8 code (*N* = 1327) was left out, 3.6% (*N* = 11,595/324,766) of all study children received ADHD diagnosis until age 12. The cumulative incidence increased from 2.8% of children born 2001 to 4.4% for 2006 born children. Association between boy sex and ADHD diagnosis increased a little in univariate and multivariate analysis when F98.8 was excluded versus included to ADHD (i.e., multivariate, boy vs. girl 5.0 [4.7–5.3] and 4.6 [4.3–4.8], respectively). In other background covariates or associations there were no significant differences.

In subgroup analyses, when all children that had received ADHD diagnoses before age 4 were left out, the cumulative incidence still 4.0% (3.9−4.0%; 12,849), in girls 1.5% (1.4−1.6; 2369) and in boys 6.3% (6.2 −6.5; 10,480). The risk of receiving an ADHD diagnosis as boy was bigger in this subgroup analysis compared to including early diagnoses. Please see Supporting Information S1: Table S8 for background characteristics.

## DISCUSSION

Consistent with our hypothesis and earlier studies (Atladottir et al., 2015; Joelsson et al., 2016; Sayal et al., 2018), we observed that the administrative incidence (diagnosed and/or treated cases) of ADHD is high in boys and is increasing more quickly for boys than girls during childhood, whereas the rate of ADHD in girls (1.9%) remained low, as in earlier studies (Dalsgaard et al., 2020; Pérez‐Crespo et al., 2020). According to healthcare data, adverse prenatal psychosocial factors were related to the risk of a child later receiving an ADHD diagnosis but may not alone explain the findings.

We found a 4.0% cumulative incidence of ADHD diagnosis in all children and compared this result to previous meta‐analyses based on diagnostic surveys of parents and teachers; the latter suggest that ADHD is more common than our findings, with a community prevalence ranging from 5.0% to 7.6% (Polanczyk et al., 2007; Salari et al., 2023; Thomas et al., 2015). However, straightforward comparisons are difficult to make due to the use of different versions of diagnostic manuals and different methodological choices (Polanczyk et al., 2007; Popit et al., 2024; Salari et al., 2023; Song et al., 2019; Thomas et al., 2015). Unfortunately, to our knowledge, no meta‐analyses on community prevalence have provided pooled estimates for children under 13 and have done so separately for boys and girls, although ADHD diagnosis is more frequent among boys during childhood (Dalsgaard et al., 2020; Huhdanpää et al., 2021; Joelsson et al., 2016; Polanczyk et al., 2007; Salari et al., 2023; Sciberras et al., 2011; Sciutto et al., 2004; Thomas et al., 2015).

Up‐to‐date estimates of the administrative incidence of ADHD diagnosis, especially via primary health care, are rare. A study on the Catalonian public healthcare register, which included 1,114,226 children and adolescents aged 4−17 years found that the prevalence of ADHD diagnoses in 2017 (defined by the ICD‐9) was 4.1% (4.0−4.1%) (Pérez‐Crespo et al., 2020), with a separate analysis showing values of 5.8% (5.8−5.9%) for boys and 2.2% (2.2−2.2%) for girls, numbers that are quite similar to our results (Pérez‐Crespo et al., 2020). A population register study using secondary healthcare data from Denmark found that the cumulative administrative incidence of ADHD diagnosis, as defined by the ICD‐10, for boys and girls born between 1995 and 2016 by age 12 was roughly 3.8% and 1.2%, respectively (based on their figures for cumulative incidence) (Dalsgaard et al., 2020). In our Finnish cohort, the difference in ADHD diagnosis between the sexes was slightly larger than in the above‐mentioned cohorts (Dalsgaard et al., 2020; Pérez‐Crespo et al., 2020). This may be due to the lower age of the participants in our study, as girls seem to receive ADHD diagnoses later than boys (Attoe & Climie, 2023; Dalsgaard et al., 2020; Mowlem et al., 2019; Pérez‐Crespo et al., 2020; Rucklidge, 2010; Westman et al., 2025). In our study, the highest incidence of ADHD diagnosis was at age eight in all children, which is in line with several earlier studies (Pérez‐Crespo et al., 2020; Rocco et al., 2021). Girls with ADHD seem to remain at risk of being underdiagnosed and undertreated in childhood, especially if the child does not show severe symptoms (Joelsson et al., 2016; Mowlem et al., 2019; Sciutto et al., 2004; Simon et al., 2009; Vuori, Koski‐Pirilä et al., 2020). ADHD in girls should be recognized at an early stage, as females with ADHD may experience more psychological, psychiatric, and cognitive distress than boys with ADHD later in life (Attoe & Climie, 2023; De Ronda et al., 2023; Skoglund et al., 2023).

In our study, ADHD diagnoses were found to be increasing, especially in boys, which poses the question of whether boys are being misdiagnosed and, if they are, the possible benefits and harms in this regard (Kazda et al., 2021). It is still unclear why the diagnosis and treatment of ADHD are more common among boys than girls during childhood (Sayal et al., 2018) and why these diagnoses are increasing. In this study, the diagnosis code of F98.8 (potentially inattentive type of ADHD) accounted for 25.4% of all ADHD diagnoses in girls and 14.2% of diagnoses in boys. When the F98.8 diagnoses were excluded, the association between male sex and the risk of ADHD diagnosis strengthened. Based on this, it is plausible to conclude that girls lacking severe hyperactivity and impulsiveness are less likely than boys with this type of ADHD to receive the F90.x diagnostic code.

We did not find any relevant increases in the studied risk factors associated with ADHD diagnosis. Therefore, the increased use of healthcare services (Sourander et al., 2008), worsened health and health habits of children, or a lower threshold for diagnosis, may be mediators of the increase in ADHD diagnosis (Centers for Disease Control and Prevention, 2010; Gyllenberg et al., 2018; Joelsson et al., 2016; Kazda et al., 2021; Polanczyk et al., 2007; Rydell et al., 2018; Sayal et al., 2018; THL, 2023; Thomas et al., 2015). We found that ADHD diagnoses in the primary healthcare register increased over the period in which the studied children were registered, which indicates that primary care clinicians are increasingly diagnosing and treating children with ADHD. Increasing numbers of ADHD diagnoses challenge healthcare and social services. For example, the use of ADHD medication for children and adolescents has increased in Finland (Kolari et al., 2024), and, in 2023, F90−F98 diagnoses became the second largest disorder group (25%), and these diagnoses have been used to justify long‐term disability benefits provided by the Social Insurance Institution of Finland (2024) and Tilastokeskus (n.d.).

Likely due to an increased awareness of ADHD and treatment options, the number of children and families with mental health and ADHD symptoms seeking services has increased in primary health care (THL, 2020), and this may have led to primary healthcare practitioners increasingly recognizing and treating ADHD. However, a comprehensive evaluation of a child with concurrent symptoms requires time, resources, and expertise, and it can be difficult to provide these resources to all regions within a country, which prior studies have confirmed (Wolraich et al., 2019). In fact, prior studies have demonstrated remarkable regional variations in ADHD diagnoses made by healthcare services (Mayne et al., 2016; Mykletun et al., 2021; Pérez‐Crespo et al., 2020; Song et al., 2019). This variation can depend on how a doctor/team reaches a clinical judgment in unclear cases, or on the available resources for psychiatric and psychosocial interventions (Mayne et al., 2016; Mykletun et al., 2021). In a recent statistical report by the Finnish Institute for Health and Welfare, a large variation was found between regions in terms of the prevalence of ADHD diagnoses (F90.x or F98.8) in 2022 in children under 13 years (from 2.0% to 20.3%) (THL, 2024). No previous studies have investigated how various regions provide services for children with mental and neurodevelopmental symptoms; thus, regional differences warrant closer evaluation.

We also studied whether prenatal and psychosocial factors were associated with ADHD diagnoses and whether these factors were associated with age at diagnosis. We found that a mother experiencing a psychiatric disorder between pregnancy and her child's fourth birthday was associated with an increased risk of her child being diagnosed with ADHD. Furthermore, as hypothesized, prenatal smoking (Ekblad et al., 2010) and moderate prematurity (Sucksdorff et al., 2015) were associated with ADHD diagnosis in childhood. These results are in line with earlier studies showing that adverse family environment factors created a larger risk of offspring developing ADHD than, for example, complications during pregnancy (Faraone, 2024). Perinatal adversities, such as socioeconomic deprivation, harsh parenting, and a maternal psychiatric diagnosis, have also been associated with ADHD diagnosis in early childhood (Hire et al., 2018; Van Batenburg‐Eddes et al., 2013; Willoughby et al., 2020; Østergaard et al., 2016). Those with early‐emerging and persistent ADHD symptoms may have a stronger hereditary background of ADHD than other groups (Franke et al., 2012; Willoughby et al., 2020). Similar to prior studies, we found a weak association between offspring receiving an ADHD diagnosis and mothers being unemployed and living alone (Willoughby et al., 2020). It is very challenging to draw conclusions regarding causal pathways within these associations, as many factors are interrelated (Bradley et al., 2001; Faraone, 2024; Mokrova et al., 2010). In an earlier Finnish cohort study, male sex, the mother's persistent depression, a negative home environment, and an authoritarian parenting style within the first 2 years after birth, in combination, increased the risk of a child being diagnosed with ADHD at age five (Huhdanpää et al., 2021). Problems in family conditions and relationships cause stress, which can be prevented and should be targeted in service planning. Small children with ADHD symptoms may require special competence to differentiate between psychological stress, developmental stage, co‐morbidities, and ADHD (Wigal et al., 2020). These multicausal problems may be associated with ADHD.

As public health care is free and available to all Finnish children and reaches, at some point, nearly all children (Sund, 2012), we believe that our results are generalizable and can be used to describe the administrative incidence of ADHD diagnosis in children. Unfortunately, the Hospital Discharge Register began to collect primary healthcare data only in 2011; therefore, some of the increase in cumulative incidence by birth cohort may be due to the late availability of that register. However, restricting the sample to children aged 10–12 years, for whom primary and secondary healthcare data were available over the entire observation period, also showed an increase in incidence by birth cohort. The role of primary health care in the diagnosis of ADHD should be studied with cohorts with longer follow‐up data in the future. In these data, the F90.0 code was the most commonly used code for ADHD diagnosis (80%), and solely with the F90.0 code, the cumulative incidence of ADHD increased from 2.5% to 4.1%. We also included unclear diagnostic codes for ADHD, such as F98.8 and F90, because these codes has been used in health care (i.e., in unclear cases or cases involving mild phenotypes of ADHD). However, we think that this methodological choice may not have caused the overestimation of ADHD diagnoses based on our sensitivity analysis results. We included all childhood ADHD diagnoses, including those received before age four (*N* = 73). As diagnostics at that age can be unreliable, we performed additional analyses to ensure reliability of the results, and offer results with and without those children. Structured diagnostic interviews or another control of diagnosis accuracy would have increased the accuracy of diagnostics but would not have been possible in this kind of setting. We did not have access to the registers of private healthcare services or data on medication purchases, which could have offered complementary information on the incidence of hyperactive disorders in children. In this study sample, maternal ADHD was very rarely diagnosed. The relationship between parent and offspring ADHD should be studied further. Unfortunately, we did not have access to data on fathers, which may have an association with children's ADHD diagnoses, although to a lesser extent than data on mothers (Huhdanpää et al., 2021). These topics and methodological choices should be addressed in future studies.

## CONCLUSION

This population register study describes the cumulative incidence and changes in the incidence of ADHD diagnoses in children up to the age of 12 in public health care from 2001 to 2018. The cumulative incidence of ADHD diagnoses increased during this period, mainly in boys. We found signs of an increase in ADHD diagnoses given by primary healthcare practitioners. However, this should be studied with longer follow‐up to conclude whether different practices in different health care sectors explain the rising numbers of diagnoses. We also found that the children of mothers with mental health issues may be at risk of being diagnosed with ADHD. However, psychosocial risk factors did not increase during follow‐up. These results demonstrate that boys and young children, especially those living in family with adverse psychosocial situation, should be diagnosed with caution to avoid misdiagnosis. Girls should be screened more often and more carefully than they currently are for all subtypes of ADHD during childhood, as they appear to face the risk of underdiagnosis.

## AUTHOR CONTRIBUTIONS

**Marika Leppänen**: Conceptualization; writing – original draft preparation; writing – review and editing; tables designing. **Miika Vuori**: Conceptualization; writing – original draft preparation; writing – review and editing; figures creation; results visualization. **Bernd Pape**: Conceptualization; writing – original draft preparation; writing – review and editing; data curation; formal analysis; software development; figures creation; results visualization. **Anniina Kaittila**: Conceptualization; writing – original draft preparation; writing – review and editing. **Siiri‐Liisi Kraav**: Conceptualization; writing – original draft preparation; writing – review and editing. **Tommi Tolmunen**: Conceptualization; writing – original draft preparation; writing – review and editing. **Merja Anis**: Conceptualization; writing – original draft preparation; writing – review and editing; funding acquisition; project administration; resource management. **Max Karukivi**: Conceptualization; writing – original draft preparation; writing – review and editing; funding acquisition; project administration; resource management. **Päivi Rautava**: Conceptualization; writing – original draft preparation; writing – review and editing; funding acquisition; project administration; resource management.

## CONFLICT OF INTEREST STATEMENT

The authors declare no conflicts of interest.

## ETHICAL CONSIDERATIONS

The study protocol was approved by the Finnish Institute for Health and Welfare (THL/2407/14.02.00/2024, on 30th in July 2024) and Turku University Hospital (J44/19, 11th in November 2019). The participants were not contacted, and informed consent was not collected, because of the study design. The legal basis for processing personal data is public interest and scientific research (EU General Data Protection Regulation 2016/679 (GDPR), Article 6(1)(e) and Article 9(2)(j); Data Protection Act, Sections 4 and 6). According to national regulations, a register‐based study does not require ethical approval or the collection of informed consent.

## Supporting information

Supporting Information S1

## Data Availability

The data that support the findings of this study are available from Finnish Social and Health Data Permit Authority Findata. Restrictions apply to the availability of these data, which were used under license for this study and data are not shared.
