# Supplementary material for: Trends in the incidence of attention deficit hyperactivity disorder in children and early risk factors
Source: JCPP Adv. 2025 Nov 12;6(3):e70067. doi: 10.1002/jcv2.70067 (PMC13339198; doi:10.1002/jcv2.70067)
Supplement: Supplementary file 1 [file JCV2-6-e70067-s001.docx]

**Trends in the Incidence of Attention Deficit Hyperactivity Disorder in Children and Early Risk Factors**

**Supporting Information**

**Table S1.** Trend Analysis of Cumulative Incidence (%) for Girls.

| Age | Birth cohort  2001 | Birth cohort  2002 | Birth cohort  2003 | Birth cohort  2004 | Birth cohort  2005 | Birth cohort  2006 | p-value |
| --- | --- | --- | --- | --- | --- | --- | --- |
| 0 | 0.00 | 0.00 | 0,01 | 0.00 | 0,01 | 0.01 |  |
| 1 | 0,02 | 0.01 | 0,01 | 0,01 | 0.02 | 0,01 |  |
| 2 | 0.02 | 0.01 | 0.01 | 0.01 | 0.03 | 0.01 |  |
| 3 | 0.05 | 0.05 | 0.03 | 0.05 | 0.06 | 0.03 | 0.228 |
| 4 | 0.08 | 0.09 | 0.08 | 0,08 | 0.08 | 0.07 | 0.881 |
| 5 | 0.18 | 0.17 | 0.15 | 0,17 | 0.17 | 0,18 | 0.964 |
| 6 | 0.31 | 0.30 | 0,27 | 0.32 | 0,29 | 0.36 | 0.512 |
| 7 | 0.42 | 0.41 | 0.46 | 0,50 | 0.53 | 0.70 | <0.001 |
| 8 | 0.54 | 0.58 | 0.69 | 0.72 | 0.83 | 0.96 | <0.001 |
| 9 | 0.69 | 0.74 | 0.87 | 0.96 | 1.09 | 1.23 | <0.001 |
| 10 | 0.84 | 0.93 | 1.07 | 1.15 | 1.31 | 1.46 | <0.001 |
| 11 | 0.98 | 1.08 | 1.24 | 1.36 | 1.55 | 1.73 | <0.001 |
| 12 | 1.12 | 1,22 | 1.39 | 1,62 | 1.83 | 1.86 | <0.001 |

**Table S2.** Trend Analysis of Cumulative Incidence (%) for Boys.

| Age | Birth cohort  2001 | Birth cohort  2002 | Birth cohort  2003 | Birth cohort  2004 | Birth cohort  2005 | Birth cohort  2006 | p-value |
| --- | --- | --- | --- | --- | --- | --- | --- |
| 0 | 0.00 | 0.00 | 0.00 | 0.00 | 0.00 | 0.00 |  |
| 1 | 0.00 | 0.01 | 0.01 | 0.00 | 0.00 | 0.00 |  |
| 2 | 0.02 | 0.01 | 0.02 | 0.02 | 0.01 | 0.02 |  |
| 3 | 0.09 | 0.06 | 0.10 | 0.10 | 0.05 | 0.09 | 0.164 |
| 4 | 0,25 | 0.25 | 0.21 | 0.27 | 0.18 | 0.26 | 0.229 |
| 5 | 0.62 | 0.63 | 0.57 | 0.60 | 0.54 | 0.63 | 0.656 |
| 6 | 1.10 | 1.23 | 1.16 | 1.13 | 1.33 | 1.35 | 0.028 |
| 7 | 1.75 | 1.79 | 1.93 | 2.18 | 2.38 | 2.68 | <0.001 |
| 8 | 2.34 | 2.53 | 2.93 | 3.26 | 3.57 | 3.94 | <0.001 |
| 9 | 2.95 | 3.24 | 3.84 | 4.29 | 4.66 | 5.13 | <0.001 |
| 10 | 3.65 | 4.00 | 4.74 | 5.13 | 5.60 | 6.20 | <0.001 |
| 11 | 4.42 | 4.76 | 5.56 | 5.89 | 6.47 | 7.31 | <0.001 |
| 12 | 5.01 | 5.32 | 6.15 | 6.60 | 7.25 | 7.70 | <0.001 |

**Table S3**. Pseudo Cumulative Incidence (%) of ADHD Diagnosis Retrieved from Primary Health Care Registers Only and Only from Age 10	upwards to ensure full coverage for all Birth Cohorts 2001-2006

| Age groups | Pseudo cumulative incidence (%) in each birth cohort | | | |  |  |
| --- | --- | --- | --- | --- | --- | --- |
| All children   10 years 11 years 12 years Sum: | Cohort 2001 | Cohort 2002 | Cohort 2003 | Cohort 2004 | Cohort 2005 | Cohort 2006 |
|  | 0.22 | 0.30 | 0.30 | 0.32 | 0.39 | 0.43 |
|  | 0.57 | 0.64 | 0.69 | 0.68 | 0.90 | 1.05 |
|  | 0.84 | 0.88 | 1.01 | 1.10 | 1.32 | 1.30 |
|  | 1.63 | 1.82 | 2.00 | 2.10 | 2.61 | 2.78 |
| Boys   10 years 11 years 12 years   Sum: |  |  |  |  |  |  |
|  | 0.38 | 0.48 | 0.48 | 0.52 | 0.65 | 0.72 |
|  | 0.95 | 1.05 | 1.13 | 1.09 | 1.47 | 1.74 |
|  | 1.39 | 1.44 | 1.67 | 1.75 | 2.09 | 2.13 |
|  | 2.72 | 2.97 | 3.27 | 3.36 | 4.21 | 4.59 |
| Girls  10 years   11 years   12 years  Sum: |  |  |  |  |  |  |
|  | 0.05 | 0.11 | 0.11 | 0.11 | 0.12 | 0.12 |
|  | 0.17 | 0.21 | 0.24 | 0.26 | 0.32 | 0.32 |
|  | 0.27 | 0.29 | 0.34 | 0.41 | 0.52 | 0.44 |
|  | 0.49 | 0.61 | 0.69 | 0.78 | 0.96 | 0.89 |

**Table S4.** Yearly incidence rates (%) of ADHD diagnoses in Boys with Full Primary Health Care Register Data Only and Cochran-Armitage tests for trend.

| Age  (years) | Cohort 2001 | Cohort 2002 | Cohort 2003 | Cohort 2004 | Cohort 2005 | Cohort 2006 | p-value |
| --- | --- | --- | --- | --- | --- | --- | --- |
| 10 | 0.38 | 0.48 | 0.48 | 0.52 | 0.65 | 0.72 | <0.001 |
| 11 | 0.56 | 0.57 | 0.65 | 0.56 | 0.83 | 1.02 | <0.001 |
| 12 | 0.44 | 0.40 | 0.54 | 0.66 | 0.62 | 0.38 | 0.17 |

**Table S5.** Yearly incidence rates (%) of ADHD diagnoses in Girls with Full Primary Health Care Register Data Only and Cochran-Armitage tests	for trends.

| Age  (years) | Cohort  2001 | Cohort  2002 | Cohort  2003 | Cohort  2004 | Cohort  2005 | Cohort  2006 | p-value |
| --- | --- | --- | --- | --- | --- | --- | --- |
| 10 | 0.05 | 0.11 | 0.11 | 0.11 | 0.12 | 0.12 | 0.024 |
| 11 | 0.11 | 0.10 | 0.12 | 0.15 | 0.20 | 0.20 | < 0.001 |
| 12 | 0.10 | 0.08 | 0.10 | 0.16 | 0.20 | 0.12 | 0.010 |

**Table S6.** Yearly Incidence Rates (%) of ADHD diagnoses in Boys with Secondary Health Care Register Data Only and Cochran-Armitage tests	for trend.

| Age  (years) | Cohort 2001 | Cohort 2002 | Cohort 2003 | Cohort 2004 | Cohort 2005 | Cohort 2006 | pvalue |
| --- | --- | --- | --- | --- | --- | --- | --- |
| 0 | 0.00 | 0.00 | 0.00 | 0.00 | 0.00 | 0.00 | 0.792 |
| 1 | 0.00 | 0.00 | 0.00 | 0.00 | 0.00 | 0.00 | 0.396 |
| 2 | 0.02 | 0.01 | 0.01 | 0.02 | 0.01 | 0.01 | 0.619 |
| 3 | 0.07 | 0.04 | 0.08 | 0.07 | 0.04 | 0.07 | 0.954 |
| 4 | 0.16 | 0.19 | 0.11 | 0.17 | 0.13 | 0.15 | 0.333 |
| 5 | 0.37 | 0.38 | 0.36 | 0.33 | 0.32 | 0.29 | 0.037 |
| 6 | 0.48 | 0.60 | 0.59 | 0.44 | 0.63 | 0.54 | 0.583 |
| 7 | 0.64 | 0.56 | 0.61 | 0.74 | 0.71 | 0.98 | <0.001 |
| 8 | 0.59 | 0.58 | 0.78 | 0.81 | 0.90 | 0.99 | <0.001 |
| 9 | 0.51 | 0.59 | 0.67 | 0.75 | 0.85 | 0.81 | <0.001 |
| 10 | 0.48 | 0,53 | 0.65 | 0.66 | 0.68 | 0.72 | <0.001 |
| 11 | 0.49 | 0,56 | 0.61 | 0.60 | 0.64 | 0.77 | <0.001 |
| 12 | 0.44 | 0,44 | 0.44 | 0.51 | 0.52 | 0.21 | 0.001 |

**Table S7.** Yearly Incidence Rates (%) of ADHD diagnoses in Girls with Secondary Health Care Register Data Only and Cochran-Armitage tests	for trends.

| Age (years) | Cohort 2001 | Cohort 2002 | Cohort 2003 | Cohort 2004 | Cohort 2005 | Cohort 2006 | p-value |
| --- | --- | --- | --- | --- | --- | --- | --- |
| 0 | 0.00 | 0.00 | 0.01 | 0.00 | 0.01 | 0.01 | 0.223 |
| 1 | 0.01 | 0.01 | 0.00 | 0.00 | 0.01 | 0.01 | 0.672 |
| 2 | 0.01 | 0.00 | 0.00 | 0.01 | 0.01 | 0.00 | 0.799 |
| 3 | 0.02 | 0.04 | 0.02 | 0.04 | 0.03 | 0.01 | 0.255 |
| 4 | 0.04 | 0.04 | 0.05 | 0.03 | 0.03 | 0.04 | 0.778 |
| 5 | 0.09 | 0.07 | 0.08 | 0.09 | 0.08 | 0.07 | 0.653 |
| 6 | 0.13 | 0.13 | 0.12 | 0.10 | 0.09 | 0.13 | 0.552 |
| 7 | 0.11 | 0.11 | 0.14 | 0.14 | 0.16 | 0.22 | <0.001 |
| 8 | 0.13 | 0.14 | 0.18 | 0.16 | 0.19 | 0.19 | 0.023 |
| 9 | 0.11 | 0.12 | 0.15 | 0.16 | 0.23 | 0.20 | <0.001 |
| 10 | 0.13 | 0.11 | 0.15 | 0.15 | 0.18 | 0.17 | 0.045 |
| 11 | 0.08 | 0.09 | 0.13 | 0.16 | 0.18 | 0.20 | <0.001 |
| 12 | 0.10 | 0.11 | 0.10 | 0.19 | 0.17 | 0.09 | 0.302 |

**Table S8.** Background factors and ADHD, when diagnosis were received at age between 4 and 12 years.

|  | First ADHD diagnosis until the age of 12 years | | First ADHD diagnosis at age of  1−7 years | | First ADHD diagnosis at age of  8–12 years | |
| --- | --- | --- | --- | --- | --- | --- |
| Odds Ratio [95% CI] | Unadjusted | Adjusted | Unadjusted | Adjusted | Unadjusted | Adjusted |
| **Mothers** (yes versus no) |  | |  | |  | |
| Smoking | 2.4 [2.3–2.5] | 2.0 [1.9–2.1] | 2.5 [2.3–2.7] | 2.0 [1.8–2.2] | 2.3 [2.1–2.4] | 1.9 [1.8–2.0] |
| Single parenthood | 1.8 [1.7–1.9] | 1.4 [1.3–1.5] | 2.0 [1.8–2.2] | 1.5 [1.3–1.6] | 1.7 [1.6–1.8] | 1.3 [1.2–1.5] |
| Non-Employed | 1.4 [1.4–1.5] | 1.1 [1.1–1.2] | 1.5 [1.4–1.6] | 1.2 [1.1–1.3] | 1.4 [1.3–1.5] | 1.1 [1.0–1.2] |
| Psychiatric disorder | 2.4 [2.3–2.6] | 2.0 [1.9–2.1] | 2.7 [2.5–3.0] | 2.2 [2.0–2.4] | 2.2 [2.1–2.4] | 1.8 [1.7–2.0] |
| First-time mother | 1.2 [1.2−1.3] | 1.0 [1.0−1.1] | 1.3 [1.2−1.4] | 1.1 [1.0−1.2] | 1.2 [1.1−1.2] | 1.0 [0.9−1.0] |
| Extra year of age at birth | 1.0 [1.0−1.0] | 1.0 [1.0−1.0] | 1.0 [0.9−1.0] | 1.0 [1.0−1.0] | 1.0 [1.0−1.0] | 1.0 [1.0−1.0] |
| **Children** |  | |  | |  | |
| Moderate preterm versus term born | 1.3 [1.2–1.4] | 1.3 [1.1−1.4] | 1.5 [1.4–1.8] | 1.5 [1.3−1.7] | 1.2 [1.1−1.3] | 1.1 [1.0–1.3] |
| Boy versus girl | 4.6 [4.3−4.8] | 4.6 [4.4–4.9] | 4.6 [4.2–5.0] | 4.6 [4.2−5.0] | 4.4 [4.1−4.7] | 4.4 [4.2–4.7] |
| **Cohort** | 1.1 [1.1−1.1] | 1.1 [1.1−1.1] | 1.1 [1.1−1.1] | 1.1 [1.1−1.1] | 1.1 [1.1−1.1] | 1.1 [1.1−1.1] |

**Figure S1.** The Cumulative Incidence of ADHD Diagnoses for Boys in Secondary Health Care.

Each birth cohort (2001−2006) was studied to determine the cumulative incidence of ADHD diagnoses with 95% Confidence Intervals; the analysis provided here is only for boys at age 0 to 12.

**Figure S2.** The Cumulative Incidence of ADHD Diagnoses for Girls in Secondary Health Care.

Each birth cohort (2001−2006) was studied to determine the cumulative incidence of ADHD diagnoses with 95% Confidence Intervals; the analysis provided here is only for girls at age 0 to 12.
